# U-survival for prognostic prediction of disease progression and mortality of patients with COVID-19

**DOI:** 10.1038/s41598-021-88591-z

**Published:** 2021-04-29

**Authors:** Janne J. Näppi, Tomoki Uemura, Chinatsu Watari, Toru Hironaka, Tohru Kamiya, Hiroyuki Yoshida

**Affiliations:** 1 3D Imaging Research, Department of Radiology, Massachusetts General Hospital and Harvard Medical School, 25 New Chardon Street, Suite 400C, Boston, MA 02114 USA; 2 Department of Mechanical and Control Engineering, Kyushu Institute of Technology, Kitakyushu, Japan

**Keywords:** Prognostic markers, Viral infection, Disease-free survival, Tomography

## Abstract

The rapid increase of patients with coronavirus disease 2019 (COVID-19) has introduced major challenges to healthcare services worldwide. Therefore, fast and accurate clinical assessment of COVID-19 progression and mortality is vital for the management of COVID-19 patients. We developed an automated image-based survival prediction model, called U-survival, which combines deep learning of chest CT images with the established survival analysis methodology of an elastic-net Cox survival model. In an evaluation of 383 COVID-19 positive patients from two hospitals, the prognostic bootstrap prediction performance of U-survival was significantly higher (P < 0.0001) than those of existing laboratory and image-based reference predictors both for COVID-19 progression (maximum concordance index: 91.6% [95% confidence interval 91.5, 91.7]) and for mortality (88.7% [88.6, 88.9]), and the separation between the Kaplan–Meier survival curves of patients stratified into low- and high-risk groups was largest for U-survival (P < 3 × 10^–14^). The results indicate that U-survival can be used to provide automated and objective prognostic predictions for the management of COVID-19 patients.

## Introduction

The rapid increase in the number of patients who have the coronavirus disease 2019 (COVID-19) has introduced major challenges for healthcare services worldwide. According to the World Health Organization (WHO), the first nine months of 2020 saw more than 34 million COVID-19 infections and more than 1 million deaths worldwide^1^, and these numbers are still increasing rapidly. Therefore, a fast and accurate clinical assessment of disease progression and mortality of patients with COVID-19 is vital for logistic planning and for management of the patients.

Recently, the WHO published specific recommendations about the use of chest imaging for the management of COVID-19 patients^2^. Chest imaging can help clinicians to decide whether to admit or discharge patients with mild COVID-19 symptoms, whether to admit patients with moderate-to-severe COVID-19 symptoms to a regular ward or an intensive care unit (ICU), and to provide information about therapeutic management of hospitalized patients with moderate-to-severe COVID-19 symptoms^2^.

Chest computed tomography (CT) is the most sensitive chest imaging method for COVID-19^3–5^; therefore, several image-based prognostic predictors have been reported for chest CT. Some of these predictors are based on quantification of the radiologist’s visual assessment of CT images^6–8^. A semi-quantitative total severity score^9,10^, which characterizes the extent of lobar involvement based on the bilateral multiple lobular ground-glass opacity and consolidation of COVID-19 on chest CT, has been shown to be predictive of patients’ mortality^7^. The extent of lung parenchyma, which can be quantified visually or by the use of image processing software for determining the volumetric size of the well-aerated lung parenchyma, has been shown to be predictive of the admission to an ICU or death^8^.

Several computer-assisted prognostic predictors have also been reported. The general approach is to extract features from CT images and to subject those features to prognostic prediction. For performing the feature extraction, deep learning^11–17^ or semi-automated image processing software^6,8,18,19^ is used for segmentation of the complete lung regions, or for segmentation of infected regions within the lungs such as ground-glass opacities, semi-consolidation, and consolidation. In studies in which complete lung regions are extracted, the segmented regions were characterized by use of radiomics with a large number of features^12,20^, whereas the segmented infected regions are typically characterized by use of a small number of well-understood manually defined features such as the relative size of a thresholded region within the complete segmented region^8,11,13–16,18,19^. In a majority of studies, the subsequent prognostic prediction has been based on logistic regression, which is largely limited to binary predictions^8,18–22^. In studies that made use of established survival analysis methodologies, the survival analysis was typically based on Cox proportional hazards regression and/or Kaplan–Meier survival curves^6,12,16^.

These previous studies on prognostic predictors for COVID-19 on chest CT had various limitations. Visual or semi-automated quantification of CT images tends to be subjective, and it can have great inter- and intra-observer variability. Only few of the automated image-based survival prediction models made use of established survival analysis methodologies, and in most studies, the analysis was limited to selected regions of interest rather than considering the complete lung region. Although some of these studies made use of deep learning, the role of deep learning has been limited to the segmentation of regions rather than using deep learning for generating new prognostic predictors^23^. Yet another limitation has been the use of manually crafted or mathematically defined features that are not necessarily ideal for the construction of optimal image-based prognostic predictors.

In this study, we present an automated image-based survival prediction model, called U-survival, which integrates the image information extracted by deep learning (U-Net) directly into a Cox proportional hazards model with an elastic-net penalty (elastic-net Cox model) for performing the prognostic prediction of patients with COVID-19. After training the U-Net to perform semantic segmentation of the lung tissue patterns of chest CT images, we subject the bottleneck section of the U-Net to an elastic-net Cox model that automatically selects a sparse subset of features to build an optimal survival model for the input data. See the subsection of “The U-survival model” under Methods for details of the design of the model.

Our approach is inspired by radiomics in the sense that we use an elastic-net penalty to construct a deep radiomic signature for survival analysis from a large number of features that are extracted from the images internally by the U-Net. Thus, we use deep learning as an integral part of the survival model, rather than as a segmentation tool. We show that the prognostic performance of the resulting U-survival model can exceed that of existing laboratory^24^ as well as visual^6,10,25^ and quantitative^8^ image-based prognostic predictors in predicting the disease progression and mortality for patients with COVID-19. We also demonstrate how the integration of an established traditional survival analysis methodology into the model makes it possible to obtain additional information, such as survival curves or risk stratification, which was not available with the previously proposed prediction models based on logistic regression. Thus, the U-survival model can be used for providing an automated and objective survival analysis with high accuracy for COVID-19 patients. Such a model could be used for logistic planning, clinical decision making, and management of COVID-19 patients.

## Results

### Summary of the dataset

We retrospectively identified 383 patients who had been confirmed as being COVID-19 positive between March 1 and June 28, 2020, at the Massachusetts General Hospital or the Brigham and Women’s Hospital (Boston, MA, USA); they were followed up until July 28, 2020. After the application of our exclusion criteria, 214 of these patients were included in the study. Table 1 shows the demographics, clinical characteristics, laboratory information, and three of the reference predictors for the 214 COVID-19 patients. Some of the information was available only for subcohorts, as indicated in Table 1.

**Table 1:** Demographics, clinical characteristics, laboratory information, and reference predictors for the patient cohort.

Variables	
Age (years)	67 [58.2, 78]
**Gender, n (%)**	
Male	128 (59.8)
Female	86 (40.2)
ICU admissions, n (%)	109 (50.9)
Deaths, n (%)	46 (21.4)
**Laboratory tests**^**a**^	
Lactic dehydrogenase (U/l)	324.0 [238.5, 438.0]
C-reactive protein (mg/l)	74.3 [35.4, 148.0]
Lymphocytes (%)	13.9 [8.6, 21.2]
Total severity score	8 [5, 13]
Percentage of well-aerated lung parenchyma^b^ (%)	69.2 [55.6, 82.1]

Continuous variables are expressed in terms of the median [interquartile range: Q1, Q3].

^a^For a sub-cohort of 175 patients.

^b^For a sub-cohort of 209 patients.

### Prognostic predictions

Bootstrap-based internal validation was performed on the U-survival model as well as on the existing prognostic predictors (hereafter called reference predictors) of (1) a combination of the laboratory tests of lactic dehydrogenase, lymphocyte, and C-reactive protein^24,26^, (2) 4D-curvature features of the CT images (abbreviated as 4DCurv)^27^, (3) the total severity score (TSS)^10,28^, and (4) the percentage of well-aerated lung parenchyma (%S-WAL)^8^.

**Figure 1:**
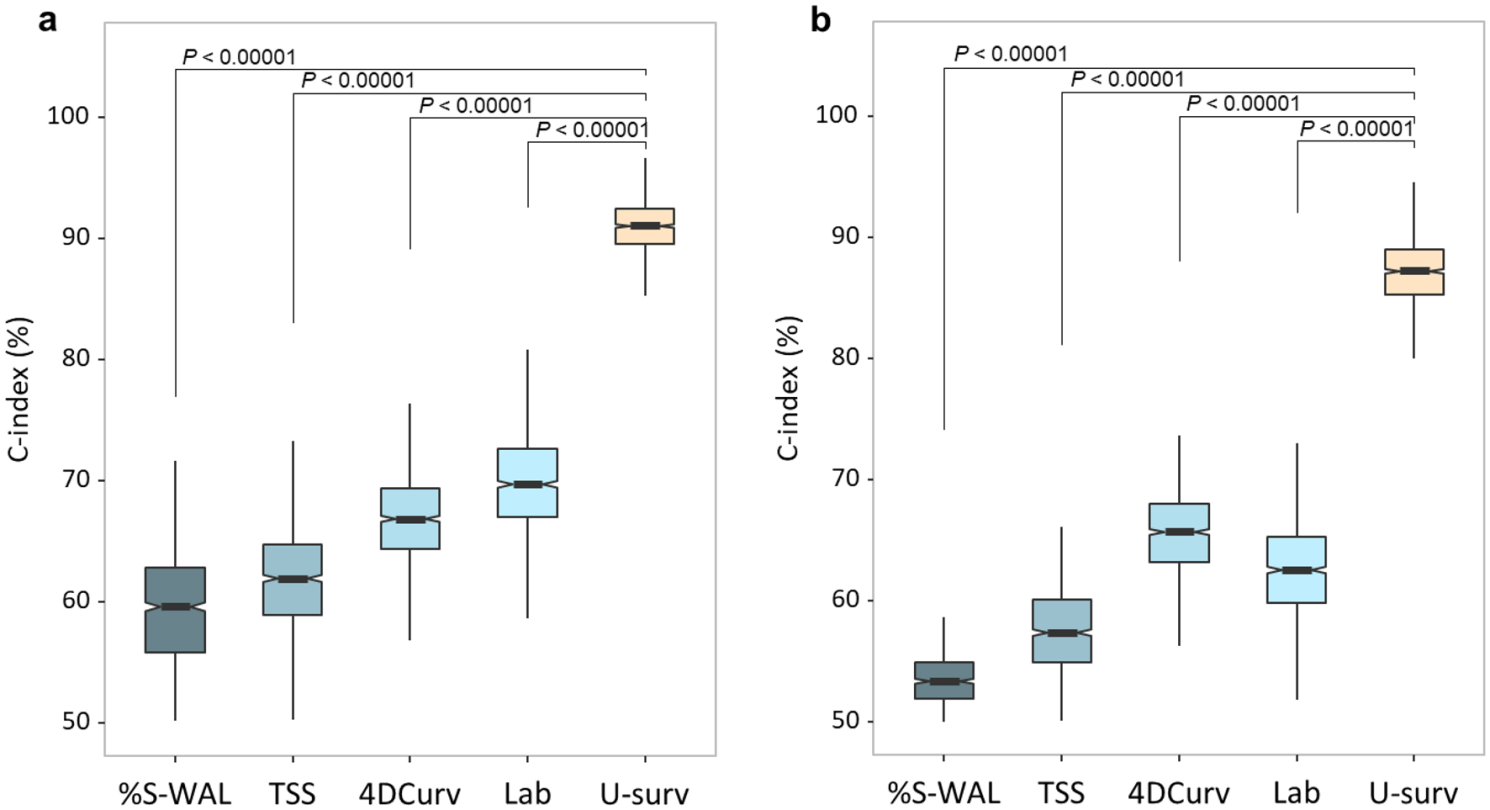
Performance of U-survival and the reference predictors in the prediction of COVID-19 progression and mortality for the subcohorts of patients for whom all the predictors were available. (**a**,**b**) Notched boxplots, in which the middle lines and the notches show the means and 95% confidence intervals, of the C-index values of U-survival and the reference predictors in the prediction of disease progression (**a**), where the outcome is defined as ICU admission or death, and in the prediction of mortality (**b**), where the outcome is defined as death. The C-index values were obtained by per-patient bootstrapping with 1000 replicates. The *P* values were obtained by use of a bootstrap hypothesis test. *%S-WAL* percentage of well-aerated lung parenchyma, *TSS* total severity score, *4DCurv* 4D-curvature, *Lab* laboratory tests, *U-surv* U-survival.

Figure 1 shows the performance of the prognostic prediction, as measured by the concordance index (C-index)^29^, of U-survival and the reference predictors in the prediction of COVID-19 (a) progression, where the outcome was defined as ICU admission or death, and (b) mortality, where the outcome was defined as death, for the subcohorts of patients for whom all of the predictors were available. The subcohort of the progression analysis in (a) included 108 patients, of whom 38 were admitted to the ICU (n = 26) or who expired (n = 12), whereas that of the mortality analysis in (b) included 173 patients, of whom 40 expired.

For the prediction of progression, U-survival yielded an estimated C-index value of 90.9% [95% confidence interval (CI) 90.8, 91.0], whereas those of the reference predictors of laboratory tests, 4DCurv, TSS, and %S-WAL were 69.8% [CI 69.5, 70.1], 66.6% [CI 66.4, 66.9], 61.8% [CI 61.5, 62.0], and 59.0% [CI 58.7, 59.3], respectively. For the prediction of mortality, U-survival showed a C-index value of 87.1% [CI 86.9, 87.3], whereas those of the reference predictors of laboratory tests, 4DCurv, TSS, and %S-WAL, were 62.5% [CI 62.3, 62.8], 65.5% [CI 65.3, 65.7], 57.0% [CI 56.8, 57.2], and 53.3% [CI 53.1, 53.4], respectively. These results indicate that U-survival outperformed the reference predictors by a large margin. The prediction performance of U-survival was statistically significantly higher than those of the reference predictors both for COVID-19 progression and for mortality (*P* < 0.00001).

Table 2 shows the performance of U-survival and the reference predictors in the prediction of the COVID-19 progression (left) and mortality (right) for the subcohorts of patients, in which all available patients (second and fourth columns) were used for each predictor. In each type of prediction and for each predictor, the C-index values were increased by 0.7–4.5% from those shown in Fig. 1. Similar to the trend shown in Fig. 1, U-survival yielded C-index value of 91.6% [CI: 91.5, 91.7] for progression and 88.7% [88.6, 88.9] for mortality, which statistically significantly (*P* < 0.00001) outperformed the other predictors by a large margin.

**Table 2:**
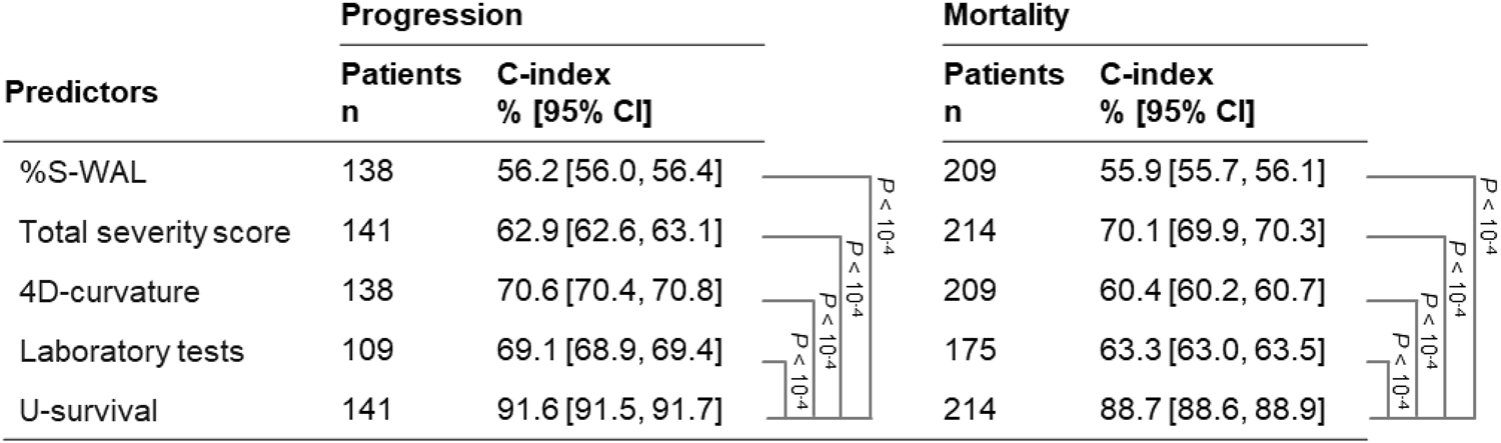
Performance of U-survival and the reference predictors in the prediction of the outcomes of COVID-19 for the subcohorts where all of the available patients were used for each predictor.

The table shows C-index values and their 95% confidence intervals (CI) obtained by per-patient bootstrapping with 1000 replicates in the prediction of disease progression (left) and mortality (right). “Patients” columns show the number of patients used for each predictor. The *P* values were obtained by application of a bootstrap hypothesis test.

*%S-WAL* percentage of well-aerated lung parenchyma.

### Risk stratification

**Figure 2:**
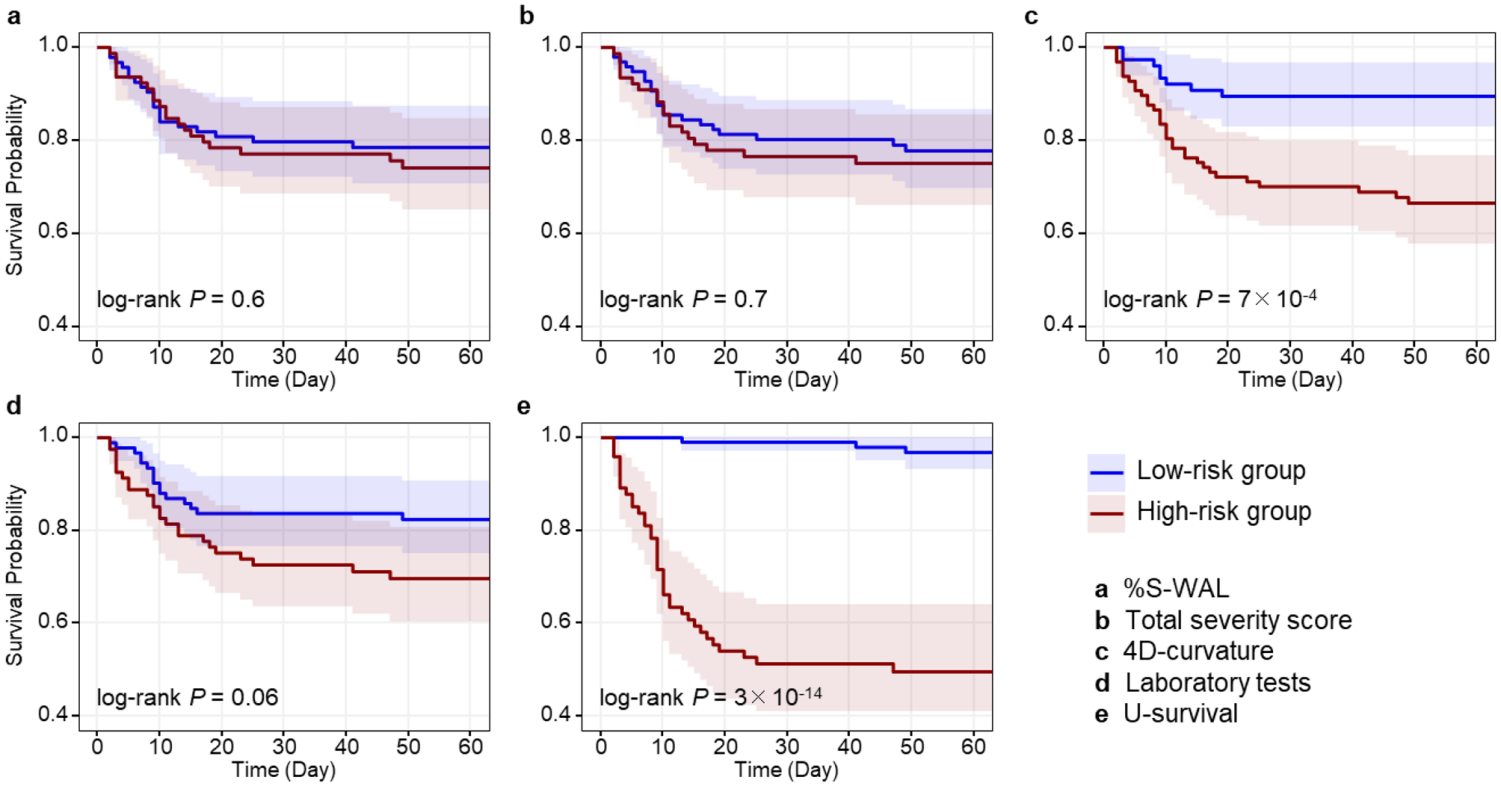
Kaplan–Meier survival curves of COVID-19 patients stratified into low- and high-risk groups based on the mortality predictions in Fig. 1b. The estimated survival curves for the low- and high-risk groups are shown in blue and red, respectively, with shaded areas representing the 95% confidence interval. The *P* values were obtained by application of the log-rank test to the two survival curves. *%S-WAL* percentage of well-aerated lung parenchyma.

Figure 2 shows the Kaplan–Meier survival curves of 173 patients stratified into low- and high-risk groups based on the mortality prediction results shown in Fig. 1b. Figure 2 indicates that the difference between the survival curves for the two groups was statistically significant for U-survival and the 4D-curvature, with log-rank *P* values of $3 \times 10^{ - 14}$ and $7 \times 10^{ - 4}$, respectively. The difference between the survival curves was not statistically significant (*P* > 0.01) for the other predictors. Visually, it is evident that the difference between the two curves was largest with U-survival, indicating that U-survival is more effective than any of the other predictors for mortality risk stratification of COVID-19 patients.

Similarly, the survival curves of 108 patients stratified into low- and high-risk groups based on the progression prediction results in Fig. 1a show that the difference between the low- and high-risk groups was largest with U-survival (Supplementary Fig. S1).

## Discussion

Although a quantitative assessment of CT images has been reported to be a predictor of disease progression and mortality for COVID-19 patients, most of the studies were based on logistic regression that is limited to the observation of binary outcomes at a given time point^30^. The use of traditional survival analysis methodologies enables the calculation of survival data as a function of time, which is desirable for the analysis of time-to-event information for applications such as the comparison of survival distributions among various risk groups^30,31^. The automated image-based U-survival model that we developed in this study is based on Cox regression analysis; therefore, it provides a continuous image-based prognostic prediction that enables us to acquire the proportional hazard for predicting the survival probability at any time point and to estimate survival curves for individual COVID-19 patients.

Our approach uses deep learning and chest CT images as an integral part of the survival prediction. Our results that greatly expand upon our previously reported methods and preliminary results^32^ show that the resulting U-survival model can substantially outperform existing laboratory tests and image-based quantitative scoring methods in the prediction of the disease progression and mortality of COVID-19 patients. An advantage of deep learning is that it is designed to learn optimal features for solving problems based on the available training data, whereas traditional manually crafted or mathematically defined image-based features are not necessarily ideal, or they are highly challenging to design for solving complex clinical problems such as the prognostic prediction investigated in this study. It should be noted that, in previous image-based prediction models for COVID-19, deep learning has been used only for the segmentation of images rather than for the extraction of prognostic features from the CT images.

In this study, we performed a systematic performance validation including the use of established performance metrics and the bootstrap method to demonstrate the generalizability of our results^35,36^. Our experiments were designed based on the international expert consensus guideline of “Transparent Reporting of a multivariable prediction model for Individual Prognosis Or Diagnosis (TRIPOD)”^35^, which provides specific recommendations regarding appropriate scientific reporting of studies involved with the development and validation of prediction models. In particular, the bootstrap method, which we used as a primary method for internal validation in this study, provides a mechanism that accounts for overfitting and uncertainty in the model development, thereby providing realistic optimism-corrected estimates of the generalization performance of the developed models^35,36^. A further advantage of bootstrapping is that the effects of predictor selection strategies on the model building, and thus the extent of model overfitting and optimism, can be quantified by repeating the predictor selection process in each bootstrap sample^35^. It has been demonstrated that the sample-based average of bootstrapping is a nearly unbiased estimate of the expected value of the optimism that would be observed in external validation^36–38^.

**Figure 3:**
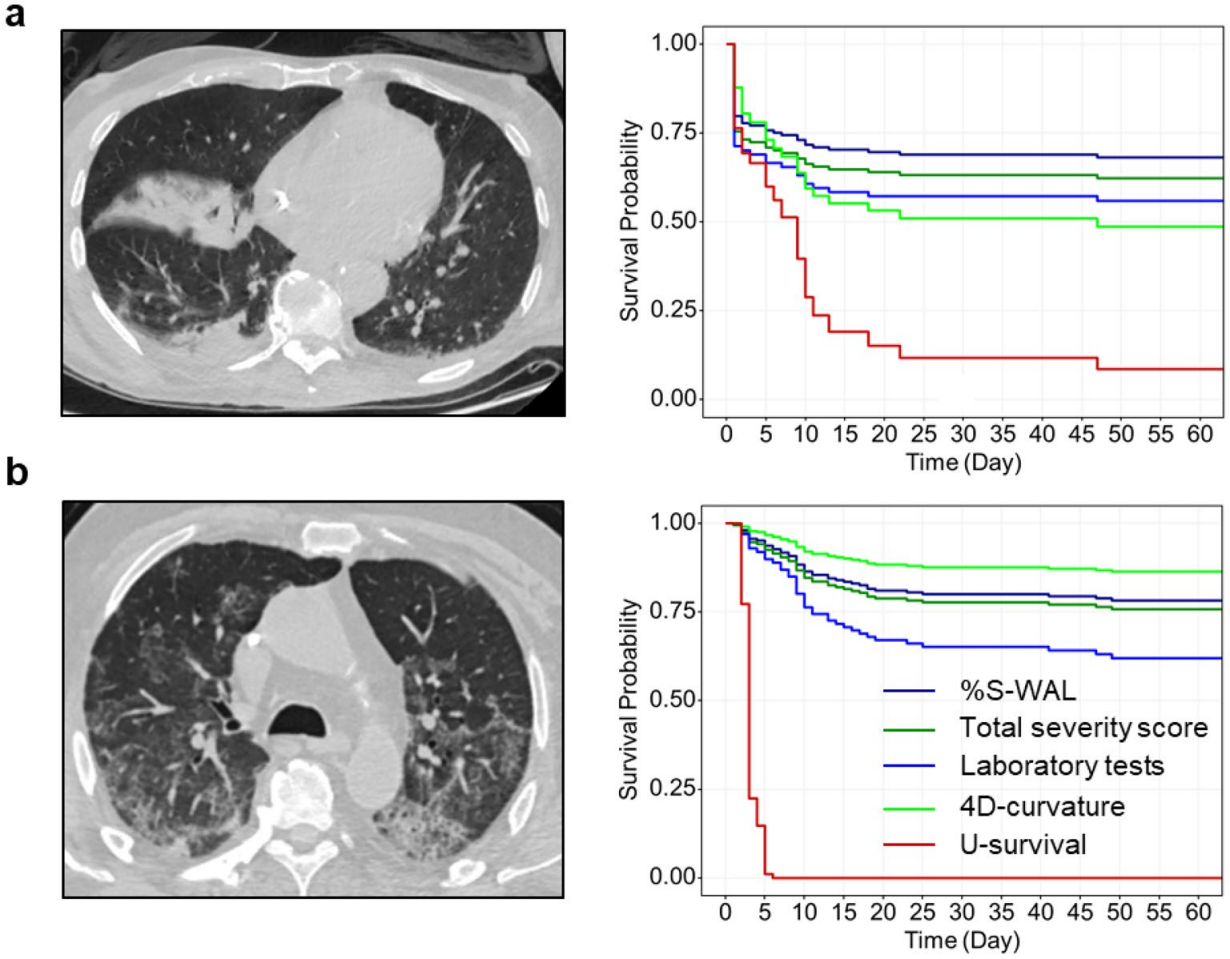
Representative chest CT images (left) and the corresponding per-patient survival curves (right) estimated by use of the predictors included in this study. (**a**) A 71-year-old male who was admitted to the ICU nine days after chest CT examination. The laboratory test results were dehydrogenase (LDH) 192 U/l, C-reactive protein (CRP) 126 mg/l, and lymphocytes 11.1%. The total severity score was 8, and the percentage of well-aerated lung parenchyma (%S-WAL) was 81.2%. The median survival times estimated by U-survival and 4DCurv were 8 and 47 days, respectively, whereas no median survival time was estimated by the other predictors. (**b**) A 64-year old male who expired three days after a chest CT examination. The laboratory test results were LDH 276 U/l, CRP 157 mg/l, and lymphocytes 4.6%. The total severity score was 12, and %S-WAL was 64.4%. The median survival time estimated by the U-survival was 3 days, whereas no median survival time was estimated by the other predictors.

Figure 3 shows two examples of the estimation of survival curves for individual COVID-19 patients by use of the prognostic predictors included in this study. For both patients, the median survival times obtained from the survival curves calculated by U-survival, as indicated by the red curve in each plot, are more accurate estimates of the observed survival time than are those of the reference predictors, demonstrating the potential usefulness of the U-survival model for the management of COVID-19 patients.

This was a retrospective study; therefore, our CT protocol was representative of routine clinical practice rather than being a carefully designed uniform study protocol. In particular, although non-contrast CT is considered sufficient for the evaluation of COVID-19 cases^33^, 57% of our cases originated from contrast-enhanced CT. In practice, contrast-enhanced CT may be used for the evaluation of COVID-19 patients who may have a clinically worsening cardiorespiratory status or suspicion of a pulmonary embolism, or for avoiding redundant imaging studies^33^. Regardless, our experiments indicated that the prognostic performance of U-survival did not vary meaningfully between non-contrast and contrast-enhanced CT (Supplementary Fig. S2).

The U-Net that we used in this study had been trained with an independent external training dataset of interstitial lung diseases for the segmentation of chest CT images into distinct lung tissue patterns. Previously, we had successfully used the pre-trained U-Net for the analysis of chest CT images of patients with idiopathic pulmonary fibrosis^34^. The use of an independent external training dataset improved the generalizability of our results, and it enabled us to maximize the number of independent COVID-19 test cases available for this study. In the future, access to a large number of CT images of COVID-19 patients could enable us to construct an independent COVID-19 dataset for the training of the U-Net, and this could yield an even higher performance of U-survival than what was reported in this study.

Our imaging study included some unique clinical information such as laboratory test results for the patients. Although CT imaging databases of COVID-19 patients are currently being made available in public imaging repositories, it is not clear whether clinical information such as laboratory tests will be included in these repositories. Thus, it may not be possible to validate our study in a larger external patient cohort any time soon.

Our results are based on the use of a 2D U-Net. In the future, it may be beneficial to use a 3D U-Net instead. However, at present, effective use of 3D deep learning is constrained by the memory limitations of currently available graphics processing units (GPUs) and by the acquisition of clinical chest CT studies at an anisotropic image resolution. Because of the GPU memory limitations, it is not possible to fit a full high-resolution chest CT volume and a sophisticated 3D deep learning model into a same GPU without the use of countermeasures such as subsampling of the image volume, which can have a detrimental effect on the performance of deep learning^39^. Because of the anisotropic image acquisition, many chest CT studies produce a stack of 2D image slices rather than a true 3D volume, and this makes volumetric analysis less meaningful than is an independent analysis of each image slice. Also, the use of 3D volumes would reduce the amount of training data in comparison to the use of 2D images. Nevertheless, in the long run, the use of a 3D U-Net with a high-capacity GPU and a large number of isotropic chest CT volumes could yield an even higher performance of U-survival than that reported in this study.

In summary, the limitations of this study as elaborated above include that this was a retrospective study and that we did not perform an external validation with an independent study population. Potential future directions include an external validation study with a large number of prospective cases and human observers from more than two institutions, an investigation of the potential benefit of a 3D U-Net in the U-survival model, and ultimately an introduction of the U-survival model to a clinical routine.

## Conclusion

We showed that deep learning of chest CT images can be used as an integral part of an automated image-based survival prediction model based on traditional survival analysis methodology. This makes it possible to obtain complete survival information that was not available with previously proposed prediction models. In our evaluation of 383 COVID-19 positive patients from two hospitals, the U-survival model significantly (P < 0.0001) outperformed existing laboratory tests and image-based visual and quantitative predictors in the prediction of the disease progression and mortality of COVID-19 patients and in the separation between the Kaplan–Meier survival curves of patients stratified into low- and high-risk groups (P < 3 × 10^–14^). The results indicate that the U-survival model can be used to provide automated and objective prognostic predictions for the management of COVID-19 patients.

## Methods

**Figure 4:**
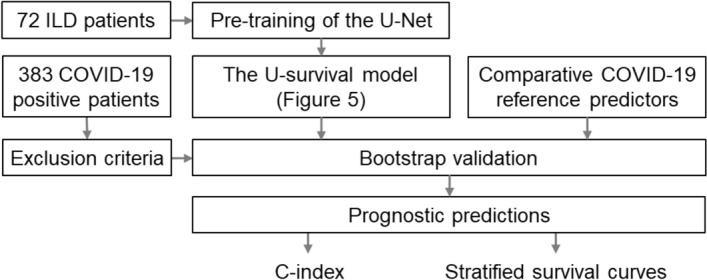
Flowchart of the key developments and evaluations. The U-survival model and the reference predictors are validated by use of bootstrapping with COVID-19 patients. The prognostic predictions are compared by use of the C-index and stratified survival curves. *ILD* Interstitial lung disease.

Figure 4 shows a flowchart of the key developments and evaluation steps described below.

### Study cohort

The study was reviewed and approved by the Mass General Brigham (MGB) institutional review board (IRB). All procedures involving human participants were performed in accordance with the ethical standards of the IRB and with the 1964 Declaration of Helsinki and its later amendments. The informed consent of the patients was waived for this study by the MGB IRB.

We retrospectively identified 383 COVID-19 positive patients between March 1 and June 28, 2020, from the medical records of the Massachusetts General Hospital and the Brigham and Women’s Hospital; they were followed up until July 28, 2020. The patients who were included in the study (1) were at least 18 years old, (2) had been diagnosed as COVID-19 positive based on a positive result for severe acute respiratory syndrome coronavirus 2 (SARS-COV-2) by reverse transcriptase-polymerase chain reaction (RT-PCR) with samples obtained from the nasopharynx, oropharynx, or lower respiratory tract, and (3) had a high-resolution chest CT examination. The resulting cohort consisted of 302 patients. After excluding the patients whose CT examinations had been performed for diseases other than COVID-19, we established a cohort of a total of 214 COVID-19 patients.

The chest CT images were acquired by use of single-phase low-dose acquisition with a multi-channel CT scanner (Canon/Toshiba Aquilion ONE, GE Discovery CT750 HD and Revolution CT/Frontier, Siemens SOMATOM Definition AS/AS + /Edge/Flash, SOMATOM Force, Biograph 64, or Sensation 64) by use of 80–140 kVp voltage, auto tube-current modulation, a pitch of 0.3–1.6, and a slice thickness of 0.625–2.0 mm. The CT images were reconstructed by use of a neutral or medium sharp reconstruction kernel.

For the patients included in this study, the mean and standard deviation of the number of days between the CT image acquisition and admission to ICU were 2.2 ± 4.1 (n = 36), whereas those between the CT image acquisition and death were 11.4 ± 10.9 (n = 46).

A subcohort of 141 patients who had a CT examination before ICU admission or who were not admitted to the ICU, was used for the prediction analysis of COVID-19 progression. For these patients, the survival time was defined as the number of days from the baseline CT image acquisition to ICU admission or death (for uncensored patients) or otherwise set to the most recent follow-up date (for censored patients). For the analysis of mortality, the entire cohort of 214 patients was used, where the survival time was defined as the number of days from the baseline CT image acquisition to death (for uncensored patients) or as the most recent follow-up date (for censored patients).

### The U-survival model

**Figure 5:**
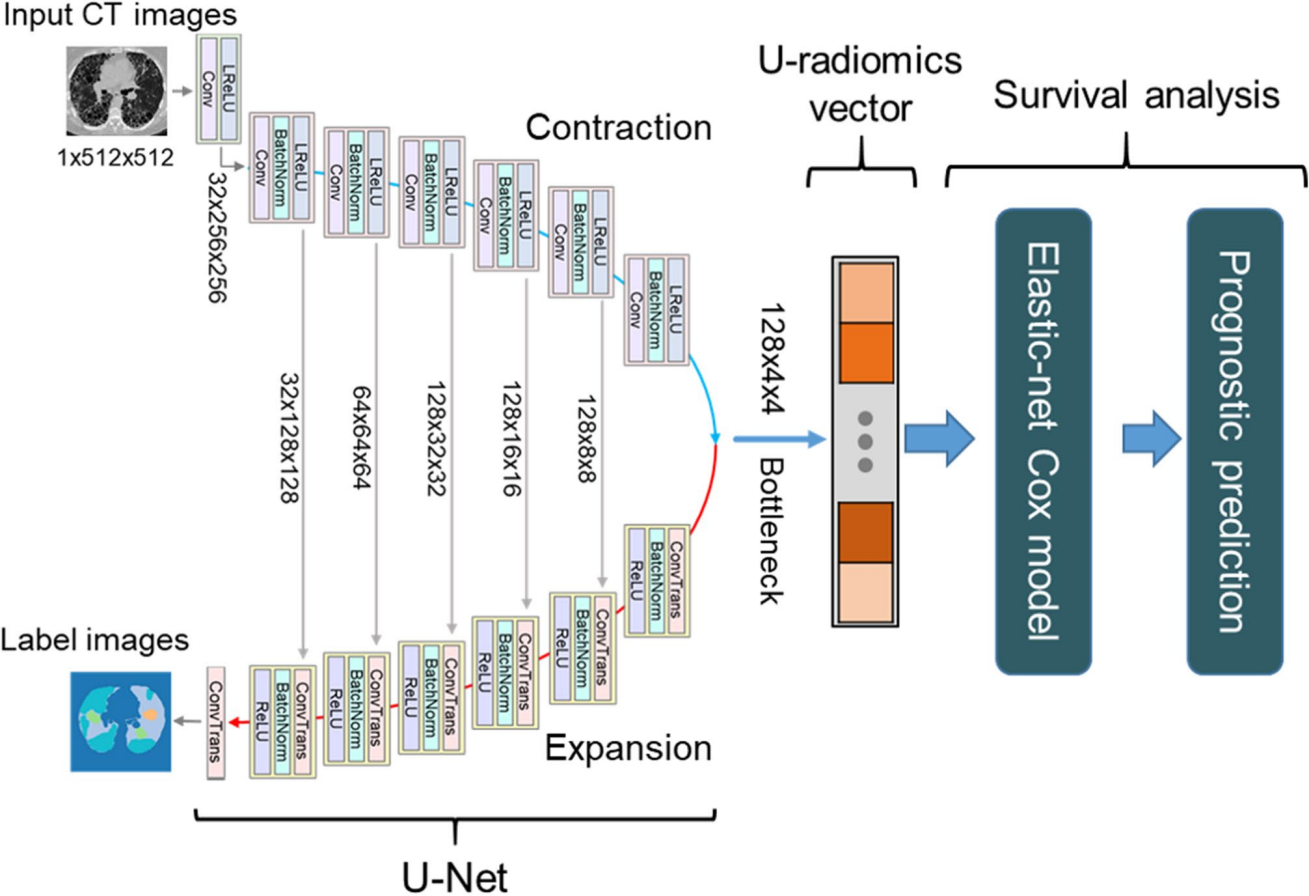
Schematic diagram of the U-survival model. The U-radiomics vector derived from the bottleneck section of the U-Net is subjected to an elastic-net Cox model for predicting the disease progression and mortality of a patient. *Conv* convolutional layer (kernel 4, stride: 2), *ConvTrans* transposed convolution (kernel 4, stride: 2), *LReLU* leaky rectified linear unit, *BatchNorm* batch normalization.

The U-survival model has two basic components: a convolutional neural network (U-Net) and an elastic-net Cox model (Fig. 5). The U-Net has three principal layer sections: contraction, bottleneck, and expansion^40^. The output of the bottleneck section that is located between the contraction and expansion sections is essentially a compressed low-dimensional representation of the input data to the U-Net.

The U-Net of this study was pre-trained, as described in our previous study^34^, for performing semantic segmentation of the lung region of axial CT images into five distinct lung tissue patterns (ground-glass opacity, reticulation, consolidation, honeycombing, and a normal healthy lung pattern) by use of the chest CT images of 72 patients who had interstitial lung diseases and corresponding tissue-pattern-labeled images (“label images” in Fig. 5). After such training, the output of the bottleneck section of the U-Net, called U-radiomics vector, contains information regarding the interpretation of the lung tissue patterns of chest CT images^41–43^.

To use this information for survival analysis, we subject the U-radiomics vector to an elastic-net Cox model^44^. This model automatically selects a sparse subset of the features of the U-radiomics vector to build an optimal survival model for the input data.

We can formulate the construction of the U-survival model as follows: Let $\{\left( {y_{n} ,{\varvec{x}}_{n} ,\delta_{n} } \right) {|} n = 1, \ldots ,N\}$ denote time-to-event data for patient $n$, where ${\varvec{x}}_{n} = \left( {x_{n}^{m} {|} m = 1, \ldots , M} \right)$ is the U-radiomics vector from the U-Net. If $\delta_{n} = 1$, $y_{n}$ is the observed survival time of an outcome such as disease progression or death for patient $n$. If $\delta_{n} = 0$, $y_{n}$ is a right-censoring time. Moreover, let $\left\{ {t_{i} | i = 1, \ldots , K} \right\}$ be an ascending list of the event times, and let $j\left( i \right)$ denote the mapping of the event time $t_{i}$ to the index of the patient at risk at that time. The construction of the Cox model involves the estimation of a hazard vector $e^{{\varvec{\beta}}} = \left( {e^{{\beta_{m} }} {|} m = 1, \ldots , M} \right)$ by maximization of the partial likelihood $L\left( {\varvec{\beta}} \right) = \prod\nolimits_{i = 1}^{K} {\left( {e^{{{\varvec{x}}_{j\left( i \right)} \cdot {\varvec{\beta}}}} /\sum\nolimits_{{j \in R_{j} }} {e^{{{\varvec{x}}_{i} \cdot {\varvec{\beta}}}} } } \right)}$, where $R_{j}$ is the set of indices for patients at risk at time $t_{i}$^45^.

In cases where the number of features in the U-radiomics vector is much larger than the number of patients (*M* ≫ *N*), the above maximum-likelihood estimation can lead to a degeneration of *β*. The elastic-net Cox model obviates this problem by use of an elastic-net regularization term.

1 $$P_{\alpha } \left( \beta \right){ } = { }\alpha \mathop \sum \limits_{i = 1}^{M} \left| {\beta_{i} } \right|{ } + { }\left( {1 - \alpha } \right)\mathop \sum \limits_{i = 1}^{M} \beta_{i}^{2} \left( {0 \le \alpha \le 1} \right)$$

where *β* is estimated by $\hat{\user2{\beta }} = {\text{argmax}}_{\beta } \left( {{\text{log }}L\left( {\varvec{\beta}} \right) - P_{\alpha } \left( {\varvec{\beta}} \right)} \right)$. The first term of Eq. (1) is the *L*1 or Lasso regularization that is known effectively to obviate the degeneration and to provide a sparse representation of *β*^46^. The second term of Eq. (1) is the *L*2 or ridge regularization, which shrinks all $\beta_{m}$ of the hazard vector to zero to obviate the degeneration, but also provides highly correlated predictors with an equal weight. When these two terms are mixed by use of the parameter *α*, the elastic-net Cox model allows the U-survival model to perform simultaneous feature selection and optimal estimation of the hazard vector $e^{{\varvec{\beta}}}$.

### Application of U-survival to CT images

To apply the U-survival model to prognostic prediction, we first apply the pre-trained U-Net to the axial CT images of a COVID-19 patient in our study cohort (see Supplementary Fig. S3). For each image, we identify the corresponding U-radiomics vector as the expanded output of the bottleneck section of the U-Net. These per-image U-radiomics vectors of the patient are then combined into a single per-patient U-radiomics vector by use of the median value of each feature of the per-image U-radiomics vectors across the axial CT images of the patient. Finally, the per-patient U-radiomics vector is subjected to the elastic-net Cox model for building a survival model for predicting the prognosis of the patient.

### Implementation and parameters of the U-survival model

The CT images of this study and those of the external training dataset for the U-Net^34^ were pre-processed for the U-Net by clipping of the intensity values to a Hounsfield unit (HU) range of − 1024 to 1024 and by mapping of these values linearly to the range of − 1 to + 1.

The free hyper-parameters of the U-survival model were optimized by use of a grid search algorithm. Specifically, the output of the bottleneck section of the U-Net was configured to be *c* × *n* × *n* = 128 × 4 × 4, where *c* and *n* represent the number of channels and the size of the output, respectively (Fig. 5). Each element of the output was post-processed by z-score normalization across the patients, and the resulting z-score normalized output was converted to a U-radiomics vector with a length of 2048. This configuration of the bottleneck section yielded 1–9% higher performance than did other possible configurations of the bottleneck section (see Supplementary Fig. S4). Similarly, we set the mixing parameter *α* in Eq. (1) to take advantage of both lasso and ridge regularization terms for optimizing the performance of the U-survival model (see Supplementary Fig. S5). For the training, we used Adam optimizer with $\beta_{1} = 0.5$ and $\beta_{2} = 0.999$. The dropout ratio was set to 0.5, batch size was 64, and the learning rate was 2.0 × 10^–4^.

### Computational environment

The U-survival model was implemented by use of our custom-made Linux-based computational server that was equipped with 10 × V100S GPUs (NVIDIA Corporation, Santa Clara, CA, USA) with 32 GB memory and 12-core 3.6 GHz Xeon Gold 6256 CPUs (Intel Corporation, Santa Clara, CA, USA) with 512 GB memory. The U-Net was implemented by use of PyTorch 1.5^47^. The elastic-net Cox model was implemented by use of glmnet 4.0^44^.

### Evaluation methods

The performance of the prognostic prediction of the U-survival model was assessed by use of the C-index^29^. The C-index is similar to the area under the receiver operating characteristic (ROC) curve (AUC) that evaluates the classification performance for binary outcomes, except that the C-index is used for estimating the concordance between predicted and observed outcomes in the presence of censoring. The C-index performs the estimation of concordance based on usable pairs, in which one patient is known to have an outcome before the other patient, who may have an outcome later or who may be censored. The C-index has a value range of 0–100%, where 50% indicates random prediction and 100% indicates perfect prediction.

To obtain optimism-corrected, generalizable estimates of the C-index values and their confidence intervals, we used per-patient bootstrapping with 1000 replications^35,38^ for the evaluation of the U-survival model. The bootstrap method enables the use of all of the available data, provides a mechanism for accounting for overfitting and uncertainty in the model development, and generates a nearly unbiased estimate of what would be observed in external validation^35,36^.

The performance of the U-survival model in risk stratification was evaluated by use of the Kaplan–Meier estimator^48^. We first calculated the optimism-corrected predictions by use of the above per-patient bootstrapping process^49^. Then, we generated the Kaplan–Meier survival curves that show the survival probability estimates over time by use of the Kaplan–Meier estimator. The survival curves are stratified into low- and high-risk groups by use of the mean of the bootstrap predictions. The difference between the survival curves between the risk groups was evaluated by use of the log-rank test, where a *P* value of less than 0.01 was considered to indicate a statistically significant difference.

The statistical analyses of the evaluation results were conducted by use of the R 4.0.2 software environment^50^.

### Comparative reference predictors

For reference, we compared the prediction performance of the U-survival model with those of several previously reported COVID-19 predictors, including (1) a combination of the laboratory tests of lactic dehydrogenase, lymphocytes, and C-reactive protein^24,26^, (2) a visual image-based CT assessment in terms of the total severity score^10,28^, and (3) a quantitative image-based CT assessment in terms of the software-based percentage of well-aerated lung parenchyma (%S-WAL)^8^. In addition, we included (4) a quantitative image-based CT assessment based on the 4D-curvature features of the CT images (4DCurv)^27^, which we previously showed to be effective in the prognostic prediction of the mortality of patients with interstitial lung diseases^51,52^.

The total severity score was obtained by a pulmonologist with 20-year experience (C.W.) based on a protocol reported in previously published studies^9,10^. Each of the five lung lobes was assessed for the degree of acute lung involvement, which was classified as none (0%) with a lobe score of 0, minimal (1–25%) with a lobe score of 1, mild (26–50%) with a lobe score of 2, moderate (51–75%) with a lobe score of 3, or severe (76–100%) with a lobe score of 4. An overall total severity score was calculated by summing of the five lobe scores (range of possible total score: 0–20). The distribution of the total severity score in the patient cohort is shown in Table 1.

The %S-WAL was calculated based on a previously published study^8^ and image processing software^27^. The software extracted the complete lung region automatically from the CT images, after which the %S-WAL was calculated as the relative volume of the well-aerated 3D lung region determined by the density interval of -950 HU and -700 HU to the volumetric size of the complete segmented 3D lung region. The distribution of the %S-WAL in the study patient cohort is shown in Table 1.

The 4DCurv characterizes each point of the complete lung region by use of hyper-curvature features of the chest CT images, including the principal curvatures, curvedness, bright/dark sheets, cylinders, blobs, and curvature scales^51^. The 4DCurv was calculated by use of previously published image processing software^27^ that generated 363 hyper-curvature features per patient^51^.

We calculated the prognostic predictions obtained from these predictors by subjecting them to the elastic-net Cox model and by applying per-patient bootstrapping with 1000 replications to the model. The C-index values obtained from the predictions were compared with those of U-survival by use of a bootstrap hypothesis test^38^, and a *P* value of less than 0.01 was considered to indicate that the difference was statistically significant.

The performances of the reference predictors in risk stratification were evaluated by use of the Kaplan–Meier survival curves that were stratified into low- and high-risk groups by the mean of the bootstrap predictions of the predictors. The difference between the survival curves for the risk groups was evaluated by use of the log-rank test, and the *P* values obtained from the test were compared with that of the U-survival model.

## Supplementary Information

Supplementary Information.

## Data Availability

The datasets used and analyzed during the current study are available from the corresponding author upon request.
