# Supplementary material for: U-survival for prognostic prediction of disease progression and mortality of patients with COVID-19
Source: Sci Rep. 2021 Apr 29;11:9263. doi: 10.1038/s41598-021-88591-z (PMC8084966; doi:10.1038/s41598-021-88591-z)
Supplement: Supplementary file 1 — Supplementary Information. [file 41598_2021_88591_MOESM1_ESM.pdf]

Supplementary Information

U-survival for prognostic prediction of disease progression and mortality of patients with COVID-19

Janne J. Näppi[+] [a], Tomoki Uemura[+] [b] [a], Chinatsu Watari [a], Toru Hironaka [a], Tohru Kamiya [b], Hiroyuki Yoshida [a]

[a] 3D Imaging Research, Department of Radiology, Massachusetts General Hospital and Harvard Medical School, Boston, MA, USA

[b] Department of Mechanical and Control Engineering, Kyushu Institute of Technology, Kitakyushu, Japan

+ These authors contributed equally to the manuscript

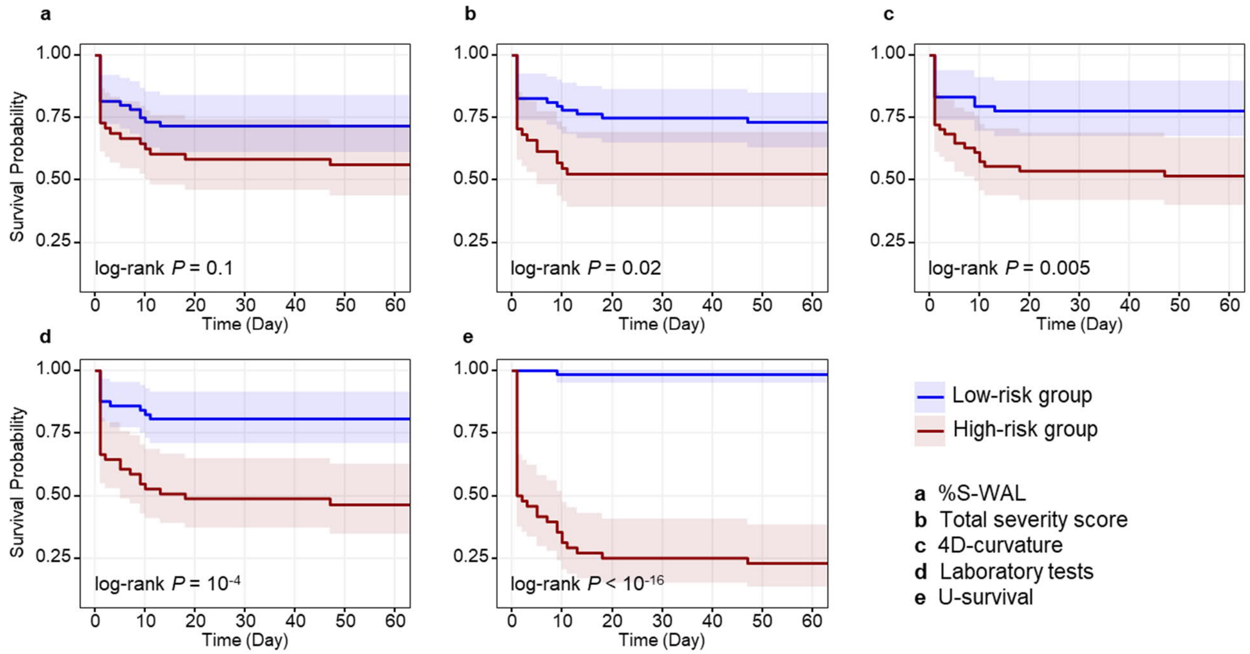

Supplementary Figure 1 | Kaplan-Meier survival curves of COVID-19 patients stratified into low- and high-risk groups based on the progression predictions of Figure 1a. The estimated survival curves for the low- and high-risk groups are shown in blue and red, respectively, with shaded areas representing the 95% confidence intervals. The P values were obtained by application of the log-rank test to the two survival curves. %S-WAL: percentage of well-aerated lung parenchyma.

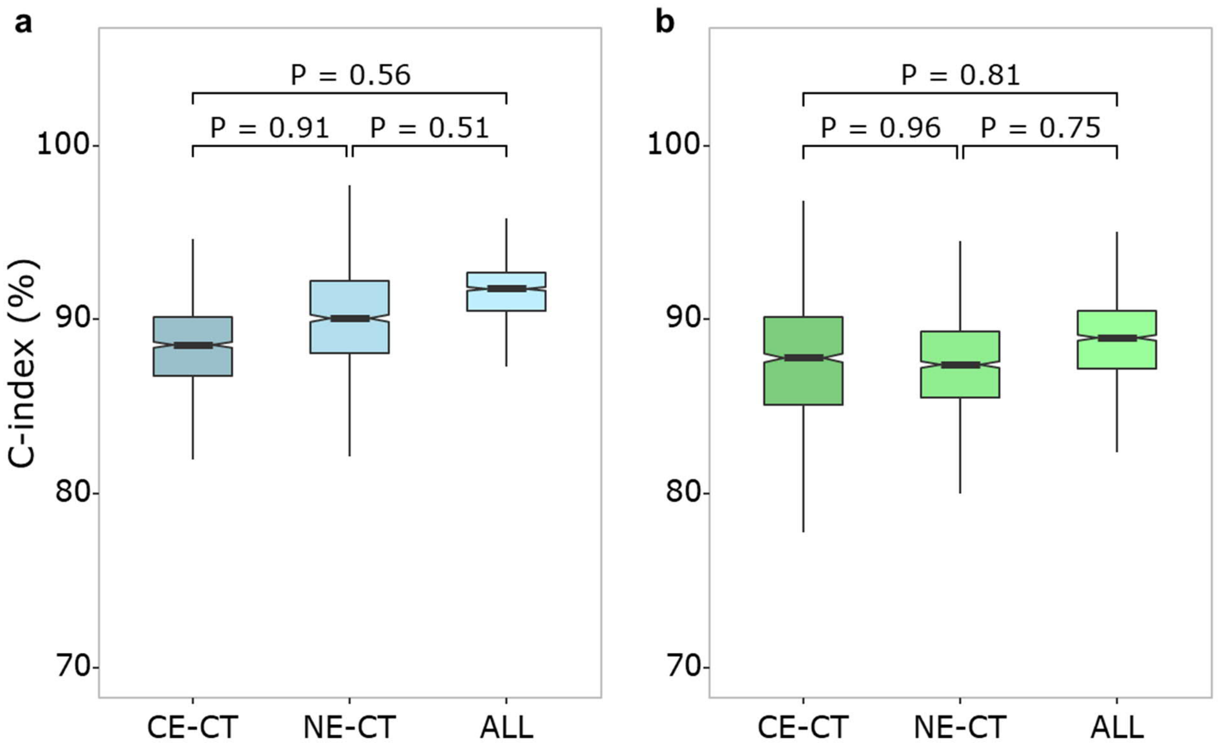

Supplementary Figure 2 | Performance differences for the U-survival model among contrast-enhanced CT (CE-CT), non-contrast-enhanced CT (NE-CT), and combined (ALL) cases. (**a**) For the 141 patients for progression prediction analysis, the U-survival model evaluated on CE-CT (88 patients), NE-CT (53 patients), and ALL (141 patients) yielded C-index values of 88.4% [95% confidence interval (CI): 88.2, 88.6], 90.0% [89.8, 90.2], and 91.6% [91.5, 91.7], respectively. The pairwise differences among these C-index values were not statistically significant (P > 0.01). (**b**) For the 214 patients for mortality analysis, the U-survival model evaluated on CE-CT (122 cases), NE-CT (92 cases), and ALL (214 cases) yielded C-index values of 87.5% [87.3, 87.8], 87.2% [87.0, 87.4], and 88.7% [88.6, 88.9], respectively. The pairwise differences among these performances were not statistically significant (P > 0.01).

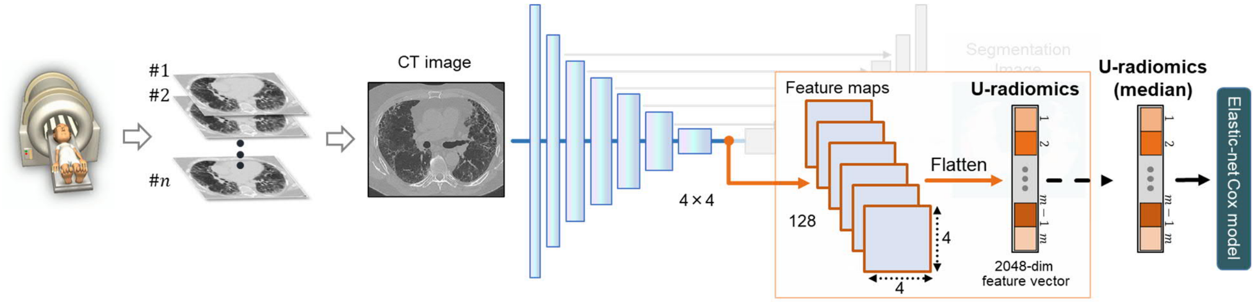

Supplementary Figure 3 | Application of the U-survival model to the CT images of a patient. After the application of the U-Net to each input CT image of a patient, the per-patient U-radiomics vector that is derived as the median U-radiomics vector of the per-image U-radiomics vectors of the patient is subjected to the elastic-net Cox model for prognostic prediction.

a

U-radiomics configuration	Progression C-index (%) [95% CI]	Mortality C-index (%) [95% CI]
128 x 1 x 1	80.6 [80.4, 80.7]	80.0 [79.8, 80.2]
128 x 2 x 2	86.5 [86.3, 86.6]	85.6 [85.4, 85.8]
128 x 4 x 4	91.6 [91.5, 91.7]	88.9 [88.8, 89.1]
256 x 1 x 1	87.2 [87.0, 87.3]	86.1 [86.0, 86.3]
256 x 2 x 2	89.9 [89.8, 90.1]	85.8 [85.6, 85.9]
256 x 4 x 4	91.3 [91.2, 91.4]	88.0 [87.8, 88.1]
512 x 1 x 1	85.9 [85.8, 86.1]	76.6 [76.3, 76.8]
512 x 2 x 2	89.8 [89.7, 89.9]	85.9 [85.7, 86.1]
1024 x 1 x 1	81.3 [81.1, 81.4]	77.0 [76.8, 77.1]
1024 x 2 x 2	88.9 [88.8, 89.0]	86.5 [86.3, 86.7]
2048 x 1 x 1	86.5 [86.3, 86.7]	82.4 [82.2, 82.6]
2048 x 2 x 2	88.5 [88.4, 88.6]	86.4 [86.2, 86.6]

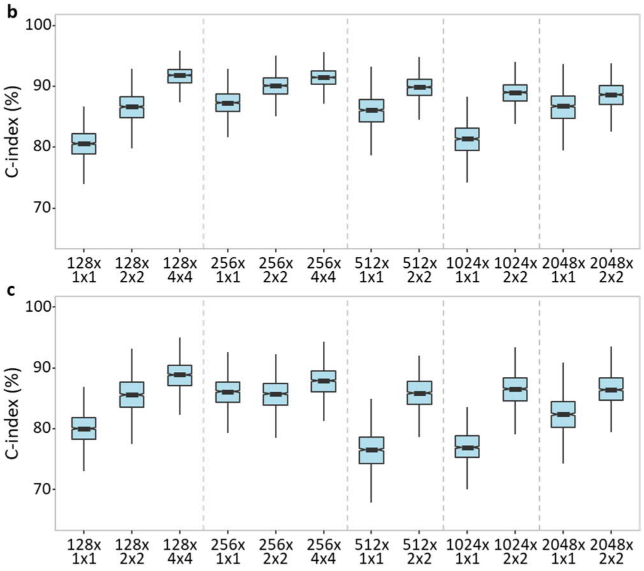

Supplementary Figure 4 | Grid search optimization of the configuration of the bottleneck section of the U-Net in the U-survival model. (**a**) The first column shows the configurations of the bottleneck section that were examined in our grid search algorithm. The second and third columns show the C-index values and their 95% confidence intervals (CIs) resulting from the bootstrap evaluation of the U-survival model for the progression and mortality analyses, respectively. (**b**) Notched boxplots of the C-index values shown in the second column of (**a**). (**c**) Notched boxplots of the C-index values shown in the third column of (**a**). In both progression and mortality analyses, the performance was highest when the bottleneck configuration was set to 128 x 4 x 4.

a

α	Progression C-index (%) [95% CI]	Mortality C-index (%) [95% CI]
0.0	90.8 [90.7, 90.9]	87.5 [87.3, 87.6]
0.1	91.8 [91.7, 91.9]	88.9 [88.8, 89.1]
0.2	91.6 [91.5, 91.7]	88.6 [88.4, 88.8]
0.3	91.4 [91.3, 91.5]	88.3 [88.1, 88.5]
0.4	91.2 [91.1, 91.4]	88.0 [87.8, 88.2]
0.5	91.1 [90.9, 91.2]	87.6 [87.4, 87.8]

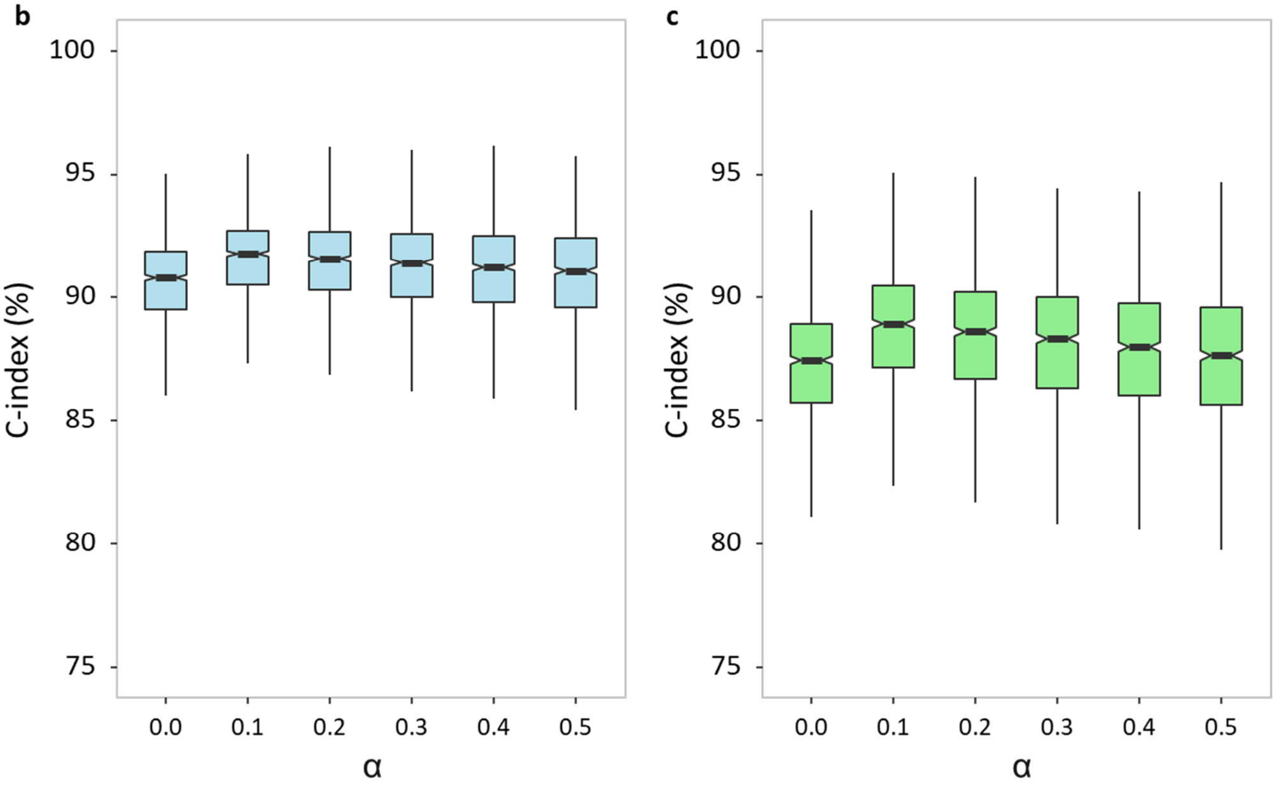

Supplementary Figure 5 | Grid search optimization of the mixing hyperparameter *α* in the elastic-net Cox model. (**a**) The first column shows the values of α that were examined in our grid search algorithm. The second and third columns show the C-index values and their 95% confidence intervals (CIs) resulting from the bootstrap evaluation of the U-survival model for the progression and mortality analyses, respectively. (**b**) Notched boxplots of the C-index values shown in the second column of (**a**). (**c**) Notched boxplots of the C-index values shown in the third column of (**a**). In both progression and mortality analyses, the performance was highest when *α* was set to 0.1.
